# Endometriosis-induced massive hemoperitoneum misdiagnosed as ruptured ectopic pregnancy: a case report

**DOI:** 10.1186/s13256-020-02486-7

**Published:** 2020-09-21

**Authors:** Bong Hyeon Kim, Seong Nam Park, Byoung Ryun Kim

**Affiliations:** 1 Department of Obstetrics and Gynecology, Wonkwang University Hospital, 895 Muwang-ro, Iksan, Jeollabuk-do 54538 Republic of Korea; 2 Department of Obstetrics and Gynecology, Wonkwang University School of Medicine, 895 Muwang-ro, Iksan, Jeollabuk-do 54538 Republic of Korea

**Keywords:** Acute abdomen, Endometriosis, Hemoperitoneum

## Abstract

**Background:**

Endometriosis, an estrogen-dependent inflammatory disease, is commonly observed in gynecologic practice. Spontaneous hemoperitoneum is a rare but serious complication of endometriosis. Most cases of endometriosis-induced hemoperitoneum are attributable to a ruptured endometrioma or utero-ovarian vessel hemorrhage. We report a case of massive hemoperitoneum secondary to intra-abdominal bleeding from the peritoneal endometriotic deposits with spontaneous abortion that was misdiagnosed as a ruptured ectopic pregnancy.

**Case presentation:**

A 36-year-old Korean woman was admitted to our hospital for acute abdominal pain and vaginal bleeding. She was suspected of ruptured ectopic pregnancy on the basis of a positive serum human chorionic gonadotropin test result and ultrasonographic evidence of pelvic fluid collection. During hospitalization, her symptoms deteriorated with peritoneal irritation sign on physical examination, hypotension, and tachycardia. Emergency exploratory laparoscopy was performed and revealed active bleeding from the peritoneal endometriotic deposit, which was treated with laparoscopic electrocoagulation. The patient’s postoperative course was uneventful. Spontaneous abortion was diagnosed on the basis of decreased serial serum human chorionic gonadotropin level estimation.

**Conclusions:**

Although rare, gynecologists should consider endometriosis-induced hemoperitoneum with spontaneous abortion in the differential diagnosis in women of reproductive age presenting with a positive serum human chorionic gonadotropin test result and acute abdomen with intra-abdominal bleeding.

## Background

Endometriosis is an estrogen-dependent inflammatory disease characterized by the deposition of endometrial tissue at extrauterine (ectopic) sites. It is a relatively common condition that mainly affects women of reproductive age [1]. Currently, retrograde menstruation, endometrial stem cell implantation, Müllerian remnant abnormalities, and coelomic metaplasia are among the several theories proposed to explain the pathogenesis of this condition; however, no single theory can conclusively explain all cases of endometriosis. Clinical manifestations of endometriosis include dysmenorrhea, dyspareunia, dyschezia, dysuria, and intermenstrual pelvic pain, as well as infertility secondary to chronic pelvic inflammation [2]. Endometriosis-related spontaneous hemoperitoneum in pregnancy and endometriosis-related ascites are rare but life-threatening complications observed in a few patients [3–5]. We report the first case of endometriosis-induced massive hemoperitoneum with spontaneous abortion that was misdiagnosed as a ruptured ectopic pregnancy.

## Case presentation

A 36-year-old gravida 1, para 1 Korean woman presented to our emergency department with a 1-day history of acute abdominal pain and vaginal bleeding. Her physical examination showed lower abdominal tenderness, and her speculum examination revealed a small amount of vaginal bleeding. Her menstrual cycle was regular at 30-day intervals, and her last menstrual period had been approximately 5 weeks prior to presentation.

Upon admission, her vital signs were stable (blood pressure 110/60 mmHg, pulse rate 64 beats/minute, body temperature 37.1 °C, oxygen saturation 99% on room air). Her laboratory test results showed mild anemia with a serum hemoglobin level of 10.1 g/dl and hematocrit of 29.0%. Her urine pregnancy and serum human chorionic gonadotropin (hCG) (1149 mIU/ml) tests showed positive results.

Transvaginal ultrasonography revealed a large amount of complex fluid and material of mixed echogenicity compatible with blood and blood clots in the pelvic cavity. The bilateral ovaries were not well visualized by ultrasonography, and a normal gestational sac was not identified in the endometrial cavity.

During her hospitalization, the patient developed pallor with dizziness, and physical examination showed positive peritoneal irritation signs along with hemodynamic instability (blood pressure 80/50 mmHg, pulse rate 110 beats/minute), necessitating emergency exploratory laparoscopy.

**Fig. 1:**
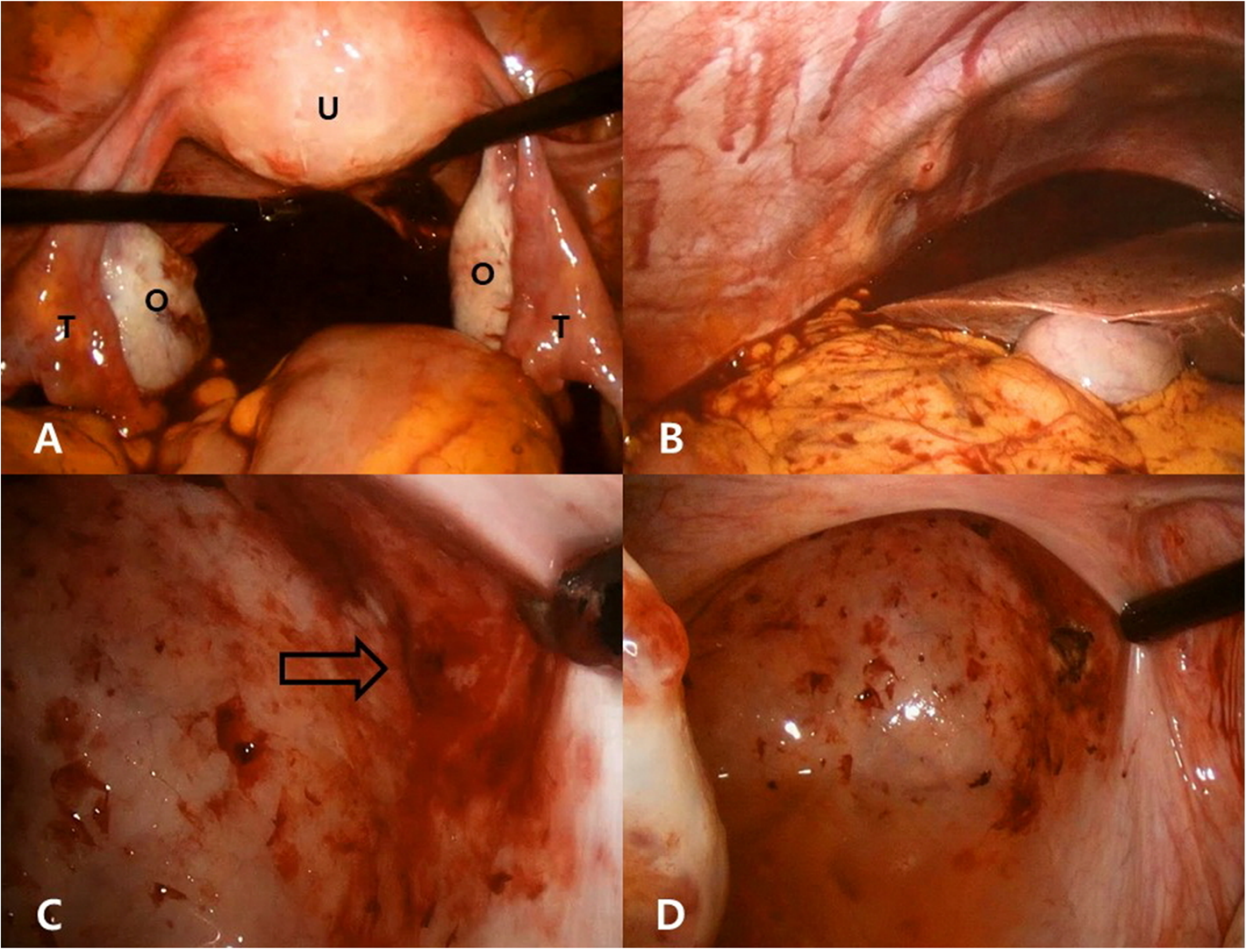
Intraoperative laparoscopic findings. **a** Fresh liquid and clotted blood in pouch of Douglas with a macroscopically normal U, bilateral O, and T. **b** Hemoperitoneum extending to the subphrenic space. **c** After suctioning the blood, active bleeding is observed from the peritoneal wall of the pouch of Douglas (*arrow*). **d** Electrocoagulation performed after excisional biopsy using a pair of laparoscopic scissors. *O* Ovaries, *T* Fallopian tubes, *U* Uterus

Laparoscopy revealed approximately 1800 ml of fresh liquid and clotted blood in the abdominal cavity. After suctioning the blood, we explored the entire abdominal cavity to identify the source of bleeding. Continuous active bleeding was observed from the peritoneal wall of the pouch of Douglas. Excisional biopsy was performed at the site of bleeding, and bleeding was controlled using electrocoagulation (Fig. 1). The intra-abdominal organs, including the uterus, bilateral ovaries, and the fallopian tubes, were inspected and appeared normal without adhesions. The patient received a transfusion of three units of red blood cells intraoperatively for hemodynamic stabilization (her serum hemoglobin level had reduced to 7.0 g/dl and hematocrit to 20.6%).

**Fig. 2:**
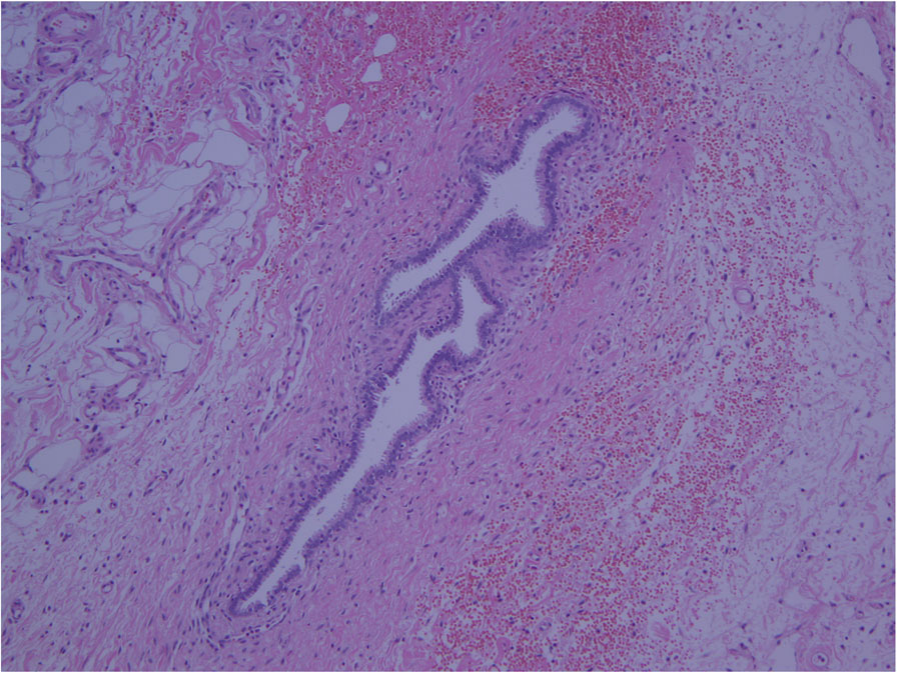
Photomicrograph showing characteristics of endometriosis with endometrial glands embedded in the stroma in the peritoneal wall (hematoxylin and eosin stain, original magnification × 100)

The patient’s postoperative course was uneventful. Her serial serum hCG levels decreased to 760.2 mIU/ml and 5.5 mIU/ml on postoperative days 1 and 13, respectively. Spontaneous abortion was confirmed on the basis of serial serum hCG level estimation. Histopathological examination of the excisional biopsy specimen revealed endometriotic deposits in the peritoneal cavity without any conceptus tissue (Fig. 2).

## Discussion

Endometriosis is a common condition observed in women of reproductive age. Patients present with a variety of symptoms, and endometriosis is diagnosed on the basis of clinical presentation, physical examination, and imaging studies (ultrasonography, computed tomography, and magnetic resonance imaging). The gold standard for definitive diagnosis remains visual inspection via laparoscopy and histopathological examination of biopsy specimens [6].

Our patient presented with vaginal bleeding and acute abdominal pain and had a positive serum hCG test result. Transvaginal ultrasonography revealed hemoperitoneum without a normal gestational sac in the endometrial cavity. Considering that she presented with a positive serum hCG test result and that her serum hCG level was less than the discriminatory level (3510 mIU/ml), we could not exclude the possibility of a normal intrauterine pregnancy despite the absence of an intrauterine gestational sac on transvaginal ultrasonography [7]. Therefore, hemoperitoneum secondary to ruptured ectopic pregnancy was the most likely preoperative diagnosis in this patient. Early normal pregnancy with intra-abdominal bleeding secondary to a ruptured corpus luteal cyst or a nongynecological condition was also considered in the differential diagnosis.

Several complications related to endometriosis that most gynecologists can overlook have been reported. Endometriosis-related spontaneous hemoperitoneum in pregnancy causes increased maternal and fetal morbidity and mortality, and endometriosis-related ascites often cause a diagnostic dilemma due to symptoms similar to an ovarian malignancy [3–5]. In addition, hemoperitoneum due to endometriosis invading extrapelvic organs such as the bowel has also been reported [8].

Acute abdomen and hemoperitoneum are rarely attributable to intra-abdominal bleeding from endometriotic deposits, and only a few cases have been reported previously in the literature. Togami *et al.* reported a case of hemoperitoneum caused by active bleeding from a peritoneal endometriotic deposit on the pouch of Douglas. The patient presented with sudden onset of lower abdominal pain from day 5 of her menstrual cycle and showed a negative serum hCG test result [9]. Mutihir and Nyango reported hemoperitoneum in a patient using progestogen for pelvic endometriosis. She had been receiving Primolut N (Bayer, Reading, UK; progestogen-only) for more than 5 years to treat endometriosis of the umbilicus and discontinued the medication for 6 days before symptom onset [10].

Our patient’s case is similar to the two aforementioned cases in that endometriosis-induced hemorrhage occurred during progesterone withdrawal [9, 10]. Therefore, it is reasonable to conclude that menstruation occurring as a result of luteolysis during the late luteal phase, abrupt cessation of progestogen-only medication, and spontaneous abortion can all precipitate a state of progesterone withdrawal and that this hormonal state contributes to hemoperitoneum secondary to active bleeding from the endometriotic deposit. However, no study in the available literature definitively clarifies the pathophysiological association between endometriosis and spontaneous hemoperitoneum.

Several studies have reported differences in the incidence of abortion between women with and without endometriosis or the possibility of reducing the incidence of abortion in women treated for endometriosis; however, convincing clinical evidence is unavailable [11]. Endometriosis-induced hemoperitoneum with spontaneous abortion has not been reported to date, and further studies are warranted to gain a deeper understanding of this condition.

## Conclusion

Gynecologists should consider hemoperitoneum secondary to intra-abdominal bleeding from endometriotic deposits with spontaneous abortion, as well as ruptured ectopic pregnancy or intrauterine pregnancy with corpus luteal hemorrhage in women of reproductive age presenting with acute abdomen and hemoperitoneum and a positive serum hCG test result.

## Data Availability

All data generated or analyzed during this study are included in this published article.

## Abbreviation

hCG: Human chorionic gonadotropin
